# Identification and Bioinformatics Analysis of the HSP20 Family in the Peony

**DOI:** 10.3390/genes16070742

**Published:** 2025-06-26

**Authors:** Haoran Ma, Heling Yuan, Wenxuan Bu, Minhuan Zhang, Yu Huang, Jian Hu, Jiwu Cao

**Affiliations:** 1 College of Forestry, Central South University of Forestry and Technology, Changsha 410004, China; mhr0078@outlook.com (H.M.);; 2 School of Foreign Languages and Tourism, Puyang Vocational and Technical College, Puyang 457000, China; yuanlin7290@163.com; 3 College of Arts, Nanning University, Nanning 530200, China; 4 College of Forestry Science and Technology, Lishui Vocational & Technical College, Lishui 323000, China

**Keywords:** peony, Hsp20 gene family, heat stress, gene expression, bioinformatics

## Abstract

Background: The peony (*Paeonia suffruticosa* Andr.), a globally valued woody ornamental species, suffers severe heat-induced floral damage that compromises its horticultural value. While the *HSP20* proteins are critical for plant thermotolerance, their genomic organization and regulatory dynamics remain uncharacterized in the peony. This study aims to systematically identify the *PsHSP20* genes, resolve their molecular features, and elucidate their heat-responsive expression patterns to enable targeted thermotolerance breeding. Methods: The genome-wide identification employed HMMER and BLASTP searches against the peony genome. The physicochemical properties and protein structures of the gene family were analyzed using online websites, such as Expasy, Plant-mPLoc, and SOPMA. The cis-regulatory elements were predicted using PlantCARE. Expression profiles under different times of 40 °C heat stress were validated by qRT-PCR (*p* < 0.05). Results: We identified 58 *PsHSP20* genes, classified into 11 subfamilies. All members retain the conserved α-crystallin domain, and exhibit predominant nuclear/cytoplasmic localization. Chromosomal mapping revealed uneven distribution without lineage-specific duplications. The promoters were enriched in stress-responsive elements (e.g., HSE, ABRE) and in 24 TF families. The protein networks linked 13 *PsHSP20s* to co-expressed partners in heat response (GO:0009408) and ER protein processing (KEGG:04141). Transcriptomics demonstrated rapid upregulation of 48 PsHSP20s within 2 h of heat exposure, with PsHSP20-12, -34, and -51 showing the highest induction (>15-fold) at 6 h/24 h. Conclusions: This first genome-wide study resolves the architecture and heat-responsive dynamics of the PsHSP20 family. The discovery of early-induced genes (PsHSP20-12/-34/-51) provides candidates for thermotolerance enhancement. These findings establish a foundation for molecular breeding in the peony.

## 1. Introduction

The peony (*Paeonia suffruticosa* Andr.), a deciduous shrub of the genus *Paeonia* (family Paeoniaceae), is a rare and endemic woody flowering species in China, renowned for its ecological value and global ornamental significance [1]. While optimally adapted to temperate-to-cool climates, its primary cultivation in China is concentrated north of the Yangtze River [2]. However, prolonged extreme heat exceeding 40 °C during summers in the regions south of the Yangtze River induces heat-induced physiological disorders, such as leaf wilting and scorching, in introduced peony cultivars [3,4,5,6], severely restricting their horticultural expansion in southern China. Consequently, elucidating the molecular mechanisms underlying heat tolerance and overcoming the ‘southward cultivation’ bottleneck have emerged as pivotal challenges in peony breeding research. Currently, transcriptomic data on the peony responses to heat stress remain scarce, and an exploration of the molecular regulatory networks demands urgent advancement [3].

Current research on heat tolerance in the peony primarily focuses on elucidating the physiological and biochemical responses alongside the molecular regulatory mechanisms [7]. Utilizing transcriptomic approaches, researchers have identified the genes and proteins responsive to heat stress in the peony [8]. For instance, an RNA-Seq-based transcriptomic database was established for the peony cultivar ‘Feng Dan’ under heat stress, enabling the screening of 237 candidate genes associated with *HSPs*, photosynthesis, oxidative stress, and ABA signaling transduction [9]. Furthermore, a gene co-expression network analysis revealed that *PsWRKY53* and *PsHsfB2b* may synergistically mediate heat stress responses in the peony [10]. Previous studies have verified and obtained some genes that can effectively enhance the heat tolerance of peonies by overexpressing them in the model plant *Arabidopsis thaliana.* Overexpression of the *PsBPM2* and *PsTRX1* genes of the peony can endow transgenic *A. thaliana* with resistance to high-temperature stress, and the interaction between the two has been verified through yeast two-hybrid (Y2H) and single luciferase complementarity assays (LCA). Similarly, overexpression of *PsDREB2A* and *PsHSFA6b* in *Arabidopsis* not only enhanced thermotolerance but significantly upregulated stress-responsive genes under heat stress [11]. The research team led by Junhui Yuan successfully deciphered the high-quality chromosome-level genome of the peony, and elucidated the molecular mechanisms underlying the formation and maintenance of its giant chromosomes. This milestone marked the entry of peony research into the genomics era, ushering in a new epoch for molecular breeding, functional characterization of elite trait-specific genes, and industrial utilization of the peony [12]. In parallel, genome-wide identification of the *NAC* gene family in the peony under heat stress revealed five heat-responsive *PsNAC* genes [13]. Leveraging whole-genome data, the researchers characterized the regulatory roles of the MYC transcription factors in peony growth and development, identifying *PsMYC2* as a key player with pronounced heat sensitivity [14].

A recent genome-wide study by Peng et al. significantly advanced our understanding by identifying 149 *HSP20* genes in peony, and revealing their statistical associations with floral organ number traits [15]. While Peng et al. provided valuable insights into the *HSP20* genes in relation to floral development, their study did not explore the potential roles of these genes in abiotic stress responses. Therefore, building upon this foundation, our study focuses specifically on the involvement of the *PsHSP20* genes in thermotolerance. We hypothesize that specific, heat-inducible *PsHSP20* genes are central mediators of heat stress adaptation in the peony [15,16]. This work aims to perform a genome-wide identification and characterization of the *PsHSP20* family, delineate their expression dynamics under controlled heat stress, and identify the key *PsHSP20* candidates contributing to heat defense mechanisms. This focus on abiotic stress aims to complement the existing knowledge and provide foundational insights for molecular breeding strategies targeting heat-resistant peony cultivar [17,18].

## 2. Materials and Methods

### 2.1. Identification of the PsHSP20 Family Genes in the Peony

The whole-genome data of the peony were retrieved from the CNSA database (https://ftp.cngb.org/pub/CNSA/data5/CNP0003098/CNS0560369/CNA0050666/, accessed on 25 June 2025). Protein sequences of the *A. thaliana* HSP20 genes (30 entries) were obtained from the TAIR database (https://www.arabidopsis.org, accessed on 25 June 2025), and the *Oryza sativa* HSP20s sequences (34 entries) were downloaded from the Gramene database. The hidden Markov model (HMM) profile of the HSP20 domain (PF00011) was acquired from the Pfam database. Using TBtools (v1. 09857) [19], a domain search against the peony proteome was performed with the HMM model (E-value < 1 × 10^−5^), generating a preliminary candidate gene set (id 1). Subsequent BlastP alignments against the *Arabidopsis* and *Oryza* HSP20 sequences (E-value < 1 × 10^−5^) yielded two additional candidate sets (id 2 and id 3). The intersection of these three gene sets was subjected to conserved domain validation via the NCBI Conserved Domain Database (CDD) and Pfam (PF00011), ultimately defining the PsHSP20 gene family members in peony.

### 2.2. Analysis of Physicochemical Properties of the PsHSP20 Family Genes

The physicochemical properties of the *PsHSP* family sequences, including coding sequence (CDS) length, amino acid length, and molecular weight, were analyzed using the Expasy online platform (https://www.expasy.org/, accessed on 25 June 2025). Subcellular localization of the *PsHSP* proteins was predicted via the Plant-mPLoc web server [19].

### 2.3. Secondary and Tertiary Structural Modeling of the Peony PsHSP20 Proteins

The secondary structures of the *PsHSP* family proteins were predicted using the SOPMA server (http://npsa-pbil.ibcp.fr, accessed on 25 June 2025), while their tertiary structures were analyzed via the Expasy platform (https://www.expasy.org/, accessed on 25 June 2025). All use the default parameters.

### 2.4. Phylogenetic Analysis of the PsHSP20 Family Genes

The amino acid sequences of the identified *HSP* family members in the peony were subjected to multiple sequence alignment using MAFFT, and the alignment results were visualized with Jalview. Phylogenetic trees were constructed in MEGA11 by integrating the *PsHSP* sequences with those of the *HSPs* from *A. thaliana*, *O. sativa*, and other species. The resulting phylogenetic trees were further annotated and refined using iTOL v6.

### 2.5. Analysis of the Structure and Domain of the PsHsp20 Genes in Peony

Conserved domains of the *HSP* family proteins in the peony were analyzed using the NCBI Conserved Domain Database (CDD), and the results were visualized with TBtools [20]. Conserved motifs were identified via the MEME Suite (http://meme-suite.org/, accessed on 25 June 2025), and subsequently visualized using TBtools [21]. Structural features of the *HSP* gene family, including intron/exon distribution, were investigated using the GSDS platform (http://gsds.gao-lab.org/, accessed on 25 June 2025).

### 2.6. Synteny Analysis and Chromosomal Localization of the PsHSP20 Family Genes

The chromosomal locations of the *HSP* genes in the peony were mapped using TBtools. Gene duplication events of the *HSPs* were identified via MCScanX using the default parameters. Interspecies syntenic relationships were visualized using the Dual Synteny Plotter tool in TBtools.

### 2.7. Analysis of Cis-Acting Elements and Transcription Factor Binding Sites in Promoter Regions

The 2-kb upstream regions of the gene structures were extracted as promoter sequences. Cis-acting regulatory elements within the promoters were predicted using the PlantCARE database, and their positions were visualized with TBtools. Transcription factor (TF) binding sites were annotated via the PlantTFDB database, and their locations were mapped and displayed on the physical promoter maps.

### 2.8. Analysis of Expression Patterns

We have already based RNA-seq sequencing in the early stage. The technology: (Illumina HiSeq™ 4000) constructed the high-temperature treated (40° C heat treated) cDNA text of the peony. The library: the original sequence is stored in the SRA (Sequence Read Archive) data library (https://www.ncbi.nlm.nih.gov/sra, accessed on 25 June 2025, login number: PRJNA1079236) [6]. Based on our previous research on the transformation of peony leaves under high-temperature stress, a protein–protein interaction (PPI) network analysis was performed on the screened *PsHSP20* genes. Gene Ontology (GO) and Kyoto Encyclopedia of Genes and Genomes (KEGG) enrichment analyses were conducted to annotate their functional roles and pathway associations. Additionally, hierarchical clustering and Pearson correlation analyses were applied to the differentially expressed *PsHSP20* genes to explore their co-expression patterns.

### 2.9. Plant Materials and Heat Stress Treatment

Using the leaves of three-year peonies as materials (‘Fengdan’), the peony plants were subjected to high-temperature stress treatment at 40 °C. Mature leaf samples were collected at 0, 2, 6, 12, and 24 h of treatment, respectively, and stored at −80 °C for RNA extraction. The specific steps referred to the method of Bu et al. [6]. The above treatments were used for all three biological repetitions.

The obtained samples were used to extract RNA and reverse transcribe it into cDNA. Real-time fluorescence quantitative PCR (quantitative real-time, qRT-PCR) was performed using the Tiangen FastReal rapid fluorescence quantitative PCR Kit (SYBR Green, Beijing, China). Primer3Plus (https://www.primer3plus.com/, accessed on 25 June 2025) was used for primer design (supplementary Table S1) and was synthesized by Anhui General Biology Co., Ltd. (Chuzhou, China). Taking the PsActin gene of the peony as the internal reference, the expression data of the candidate genes were obtained based on fluorescence quantitative PCR. The gene expression levels were calculated using 2^−ΔΔCt^. The experiment was repeated three times. The specific steps referred to the method of Bu et al. [6]. The gene expression data were analyzed by student-t difference test with the help of IBMSPSS 20.0 software. A *p* value < 0.05 indicated a statistically significant difference.

## 3. Results

### 3.1. Genome-Wide Identification and Physicochemical Characterization of the PsHSP20 Gene Family in the Peony

Candidate genes were screened based on the conserved domains of the *HSP20* genes from *Arabidopsis* and *Oryza*, resulting in the identification of 58 *HSP20* family genes in the peony, designated as *PsHSP20-1* to *PsHSP20-58*. The *PsHSP20* proteins show significant physicochemical diversity: the amino acid lengths of the *PsHSP20* proteins ranged from 134 to 359, the isoelectric points (pI) varied from 4.85 to 9.74, and the instability index ranged between 27.18 and 73.75, while the hydrophobicity coefficient (GRAVY) values ranged from 50.18 to 109.82. Except for *PsHSP20-8* (hydrophilicity coefficient: 0.06), all proteins were hydrophilic, with *PsHSP20-1* exhibiting the strongest hydrophilicity (−0.982). Subcellular localization predictions indicated that 49 proteins were localized to the cytoplasm/nucleus, 5 to mitochondria, *PsHSP20-35* and *PsHSP20-56* to peroxisomes, *PsHSP20-44* to chloroplasts, and *PsHSP20-45* to the extracellular matrix (Supplementary Table S2).

### 3.2. Structural and Conserved Domain Analysis of the PsHSP20 Genes

**Figure 1:**
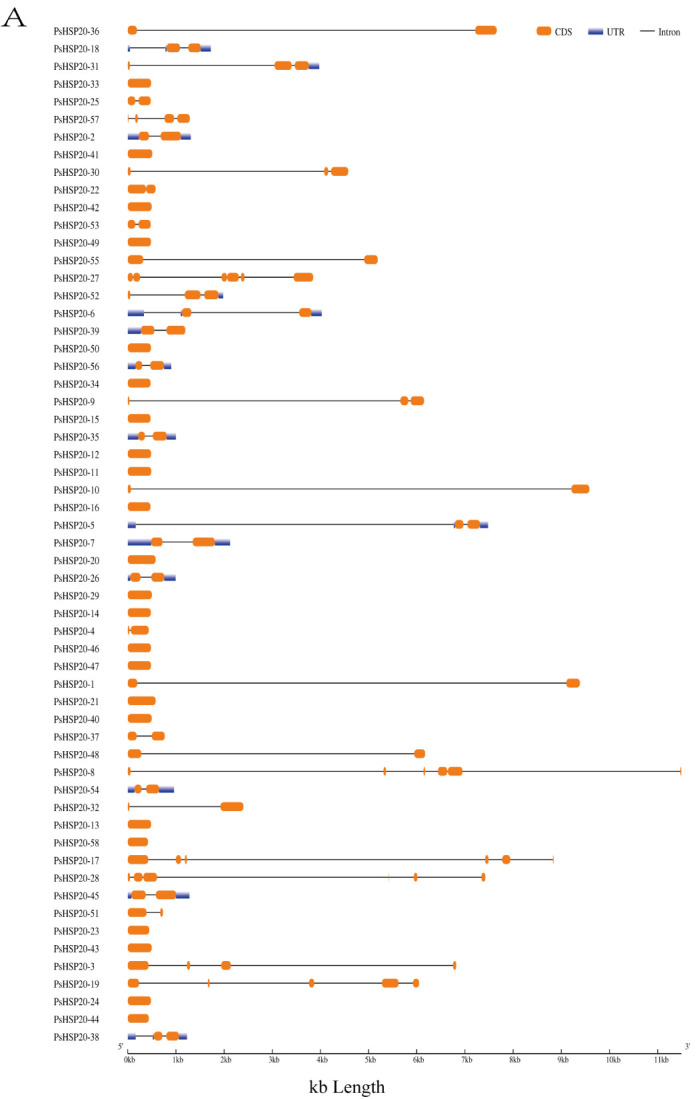

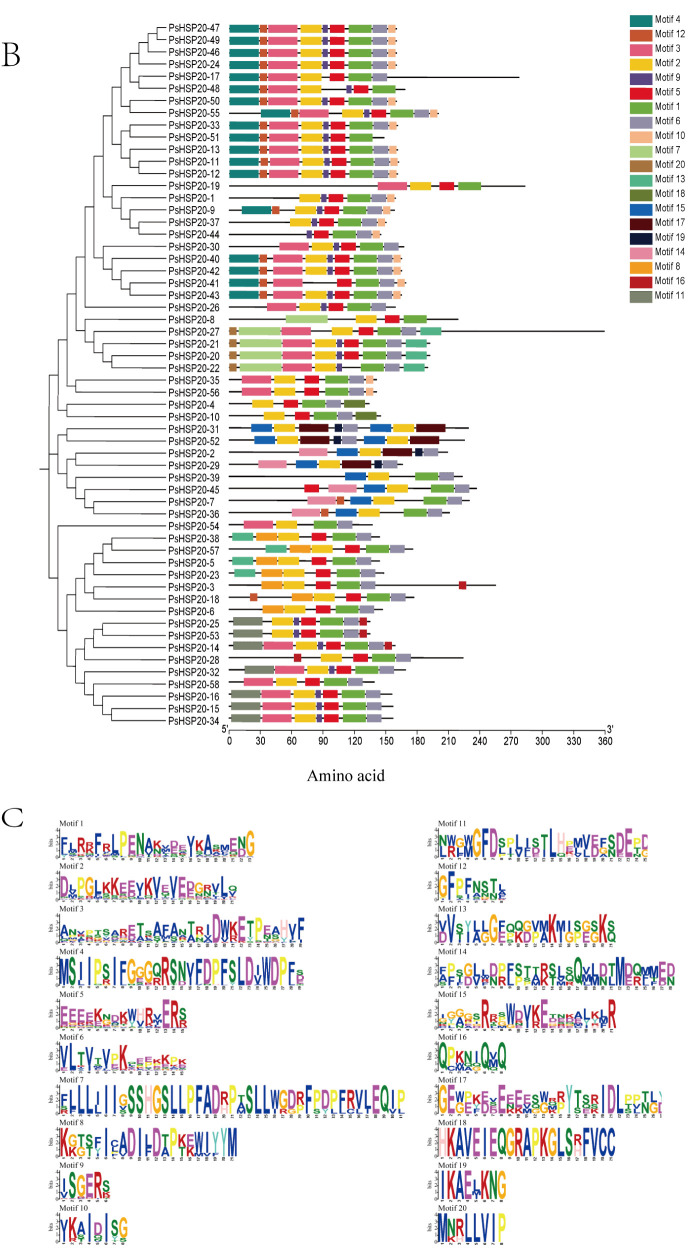
Structural and motif analysis of the PsHSP20 gene family in the peony: (**A**) Exon–intron structure of PsHSP20 genes; (**B**) Distribution of conserved motifs (Motif1–Motif20) identified in PsHSP20s proteins. Motifs are color-coded; (**C**) Sequence logos of Motif1 and Motif2, representing the core conserved domains of PsHSP20s.

A gene structure analysis revealed a predominantly intron-poor architecture in the *PsHSP20* family, with 72.4% of the genes (43/58) containing ≤ 1 intron. Conversely, only 8.6% (5/58) exhibited complex structures harboring ≥ 4 introns (Figure 1A). The *PsHSP20* family encompasses 20 conserved motifs (Motif1–Motif20), ranging in length from 6 to 41 amino acids. Motif1 and Motif2 were the most prevalent across the *PsHSP20* sequences, suggesting their roles as core conserved domains. Phylogenetic tree clustering further indicated that *PsHSP20* members within the same clade shared similar Motif arrangements. For example, the CVII subfamily exhibited a motif order of Motif15, Motif2, Motif17, Motif19, Motif6, Motif15, Motif2, and Motif17, whereas the Po subfamily displayed the distinct pattern of Motif3, Motif2, Motif5, Motif1, Motif6, and Motif10 (Figure 1B,C). These findings demonstrate subtle structural variations among the *PsHSP20* members from different subfamilies.

### 3.3. Secondary and Tertiary Structural Features of the PsHSP20 Proteins

**Figure 2:**
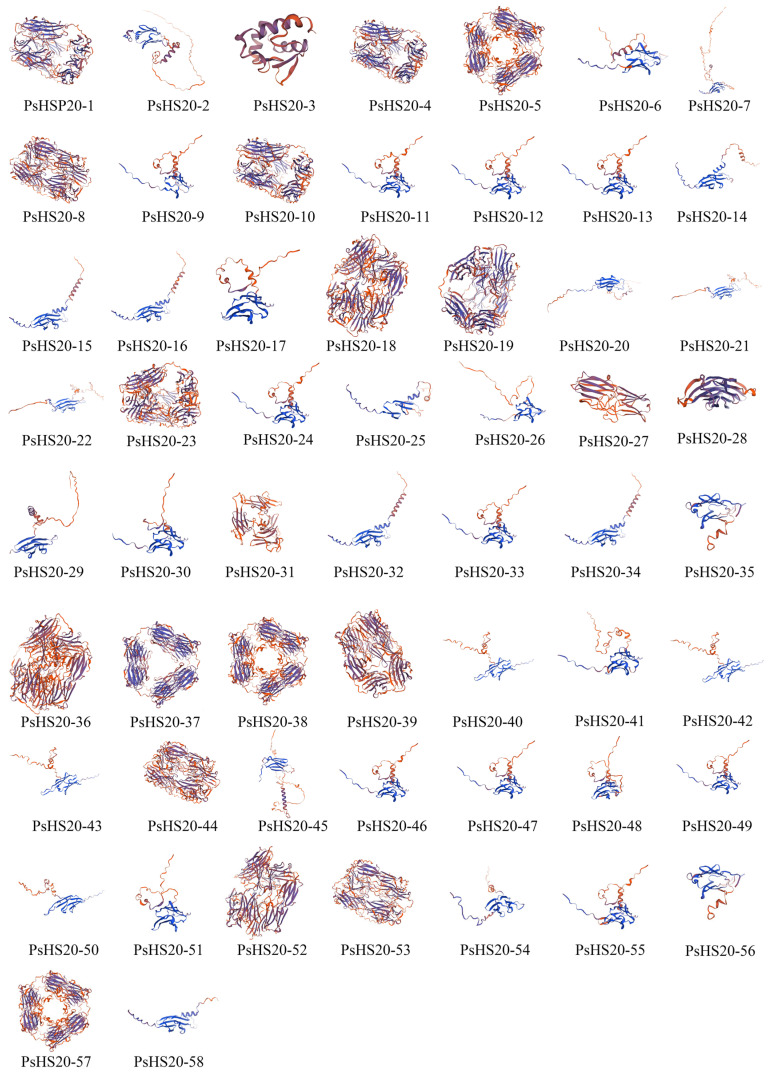
Predicted tertiary structures of the *PsHSP20* proteins in the peony. Three-dimensional models were generated using SWISS-MODEL.

The secondary structure analysis of the *PsHSP20* proteins is summarized in Supplementary Table S3. The secondary structures of the *PsHSP20* family are composed of four components: α-helices, extended strands, β-sheets, and random coils. Among them, *PsHSP20-28* exhibited the highest proportion of α-helices (31.70%), while *PsHSP20-4* showed the largest proportion of extended strands (26.12%). *PsHSP20-53* displayed the highest β-sheet content (7.41%), and *PsHSP20-31* had the highest proportion of random coils (64.19%). The tertiary structures of the *PsHSP20* proteins were predicted using the SWISS-MODEL server (Figure 2). The results demonstrated that 44 *PsHSP20* tertiary structures could be clustered into 10 groups (2–11 members per group), with high structural similarity within each group, suggesting potential functional conservation. The remaining 24% of the tertiary structures showed no significant similarity.

### 3.4. Chromosomal Localization and Synteny Analysis of the PsHSP20 Genes

**Figure 3:**
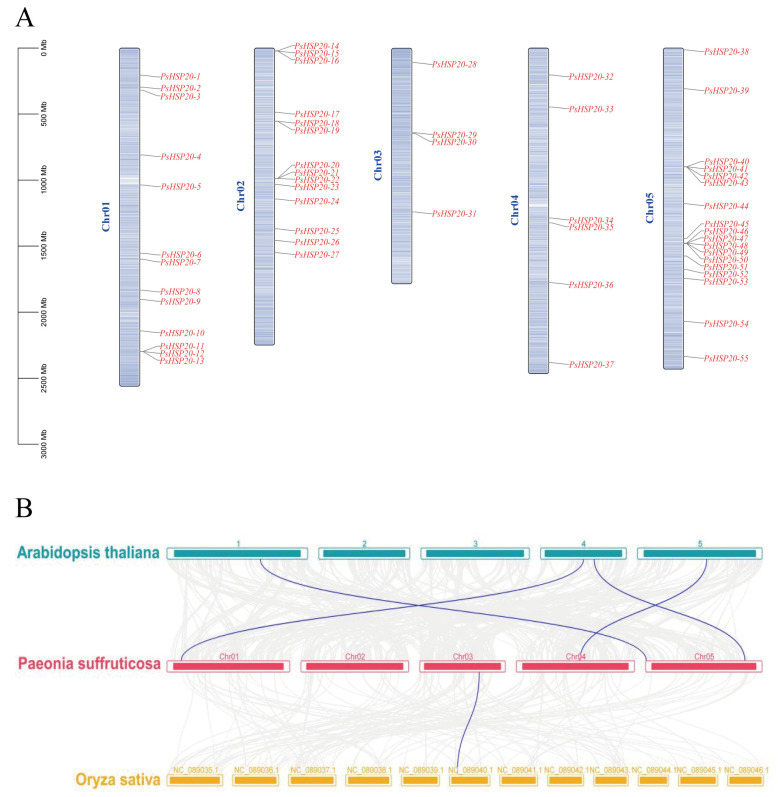
Chromosomal localization and synteny analysis of the *PsHSP20* genes: (**A**) Chromosomal distribution of 55 *PsHSP20* genes across five chromosomes (Chr01-Chr05). Gene positions are marked with vertical lines; (**B**) Syntenic relationships between *PsHSP20* genes and orthologs in *A. thaliana* (At) and *O. sativa* (Os). Colored lines indicate homologous gene pairs.

The chromosomal localization of the *PsHSP20* family was visualized using TBtools (Figure 3A). Among the 58 genes, 55 were unevenly distributed across all five chromosomes of the peony, while *PsHSP20-56*, *PsHSP20-57,* and *PsHSP20-58* remained unanchored to chromosomal scaffolds. The start and termination positions of these genes are detailed in Supplementary Table S4. Chromosome 3 (Chr03) harbored the fewest *PsHSP20* genes (*n* = 4), whereas Chr05 contained the highest number (n = 18), with genes predominantly clustered in its mid-to-late regions. A synteny analysis between the peony, *Arabidopsis*, and *Oryza* (Figure 3B) revealed only one homologous gene pair between the *PsHSP20s* and the *OsHSP20s*, but four orthologous gene pairs between the *PsHSP20s* and the *AtHSP20s*, indicating a closer evolutionary relationship between the *HSP20* families of the peony and *Arabidopsis*. An MCScanX analysis detected no gene duplication events within the *PsHSP20* family.

### 3.5. Phylogenetic Classification and Evolutionary Relationships of the PsHSP20 Genes

**Figure 4:**
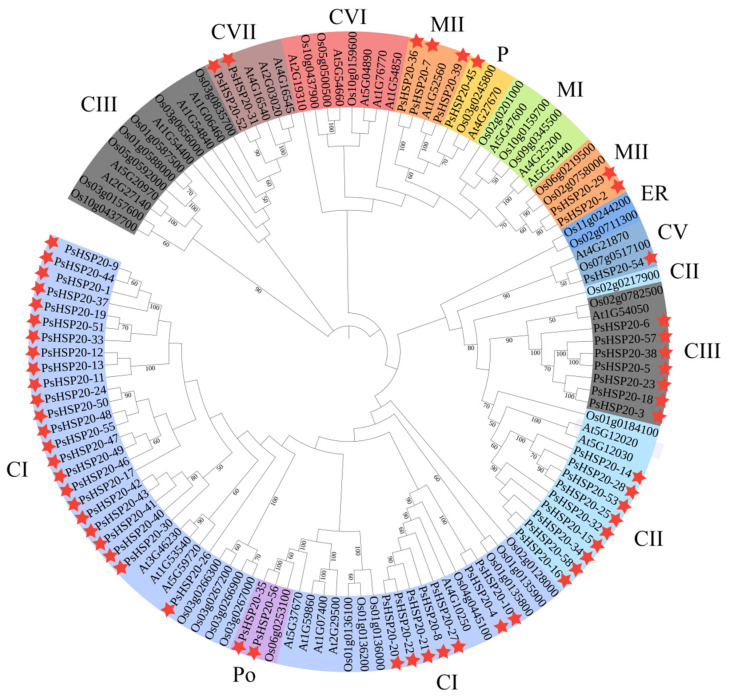
Phylogenetic analysis of the *HSP20* proteins in the peony, *A. thaliana* (At), and *O. sativa* (Os). The maximum likelihood tree was constructed using MEGA11 with 58 *PsHSP20*, 30 *AtHSP20*, and 34 *OsHSP20* sequences. Branches are colored to represent the 11 subfamilies (CI, CII, CIII, CV, CVI, CVII, MI, MII, P, Po, and ER). The stars indicate the genes for association analysis.

To elucidate the evolutionary relationships within the *HSP20* family, we constructed a phylogenetic tree by comparing sequences from the peony with those of the other species. A total of 122 protein sequences—30 *AtHSP20s* from *Arabidopsis*, 34 *OsHSP20s* from *Oryza*, and 58 *PsHSP20s* from the peony—were analyzed using MEGA10 to generate the phylogenetic tree (Figure 4). The phylogenetic analysis classified the *PsHSP20s* into 11 subclades (CI, CII, CIII, CV, CVI, CVII, MI, MII, P, Po, and ER). Among them, the cytoplasmic CI is the largest subfamily, and the plastid P subfamily is the smallest, containing only one peony *PsHSP20-45* gene. Clustering results indicated that most *PsHSP20* proteins function in the cytoplasm. The tree topology revealed a close evolutionary relationship between the plastid P-group and the mitochondrial MI/MII subclades, consistent with the prior findings that the mitochondrial M-group of the *HSP20* family evolved from the plastid P-group [18]. Notably, the *PsHSP20* family exhibited a significantly larger gene repertoire compared to the *OsHSP20* and *AtHSP20* families, with closer phylogenetic affinities to *Arabidopsis* than to *Oryza*.

### 3.6. Promoter Architecture: Cis-Acting Elements and Transcription Factor Binding Sites

**Figure 5:**
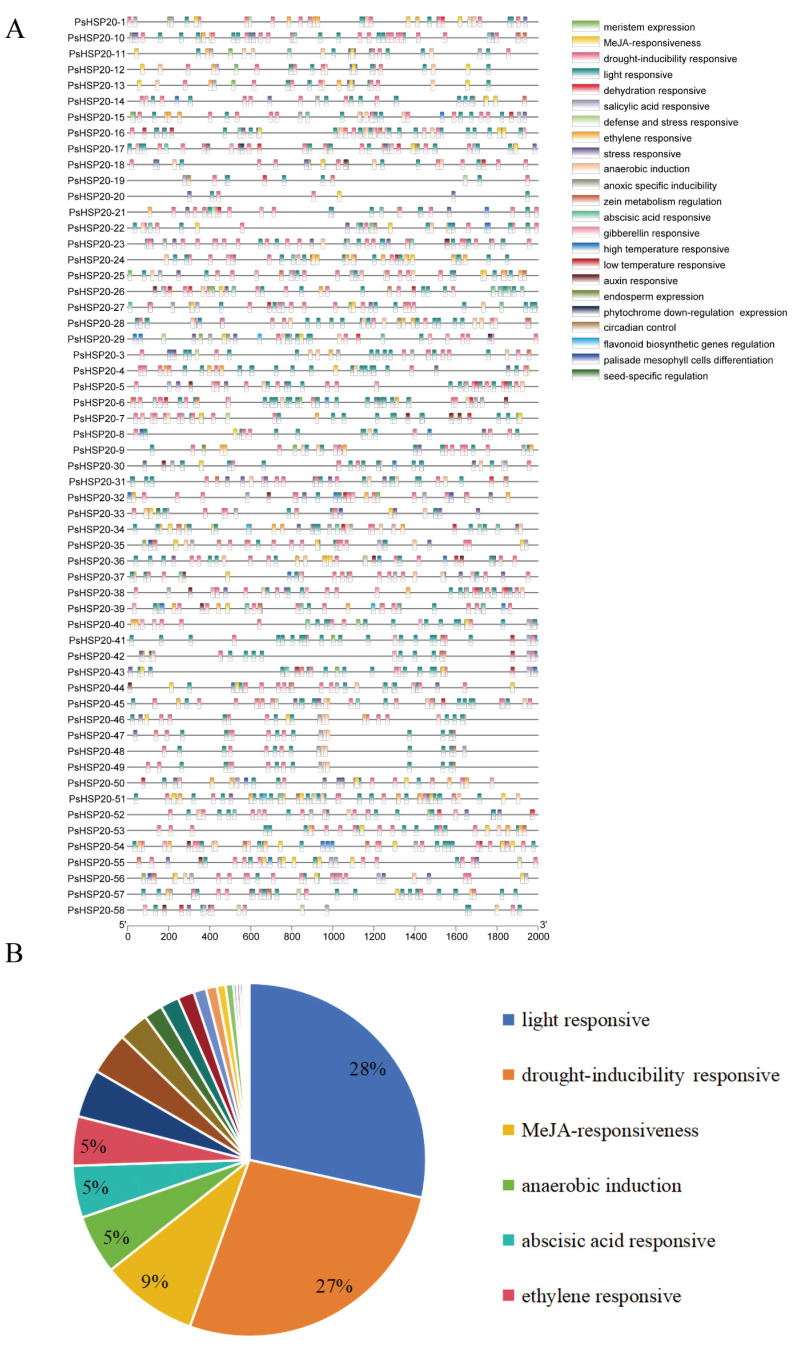
Cis-acting regulatory elements in the promoter regions of the *PsHSP20* genes: (**A**) physical map of cis-elements in the 2-kb upstream regions of the PsHSP20 genes; (**B**) proportion of cis-element categories, including light-responsive (28%), drought-responsive (27%), and heat-responsive elements (highlighted in red).

**Figure 6:**
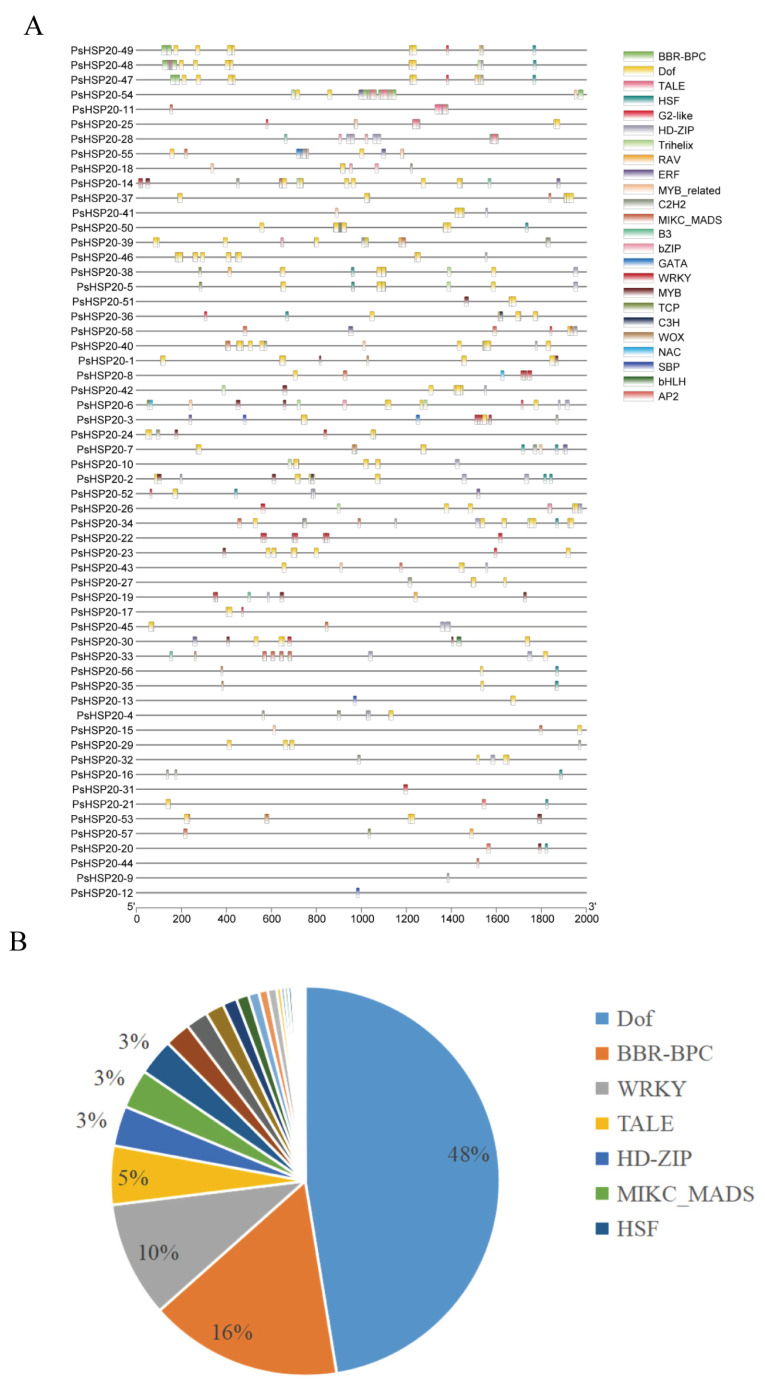
Transcription factor (TF) binding sites in the promoter regions of the PsHSP20 genes: (**A**) physical map of the TF binding sites in the promoter regions; (**B**) proportion of TF families, with Dof (642 sites) and BBR-BPC (217 sites) being the most abundant.

The 2000-bp upstream regions of each of the *PsHSP20* genes were analyzed for cis-acting elements (Figure 5A; Supplementary Figure S1), identifying 23 categories of regulatory elements. Notably, light-responsive (28%) and drought-responsive (27%) elements were significantly enriched, suggesting broad involvement of this family in abiotic stress and hormonal signaling pathways (Figure 5B). Heat-responsive elements were detected in only 24 genes, with *PsHSP20-54* harboring the highest number, implicating it as a central hub in heat stress regulation. A TF binding site analysis (Figure 6A) predicted 24 classes of motifs in promoter regions, with the Dof (642 sites) and BBR-BPC (217 sites) families being the most abundant (Figure 6B). Among the key genes, *PsHSP20-48* and *PsHSP20-50* contained the highest number of TF binding sites (Supplementary Figure S2), while *PsHSP20-6* exhibited the greatest diversity of binding motifs. These findings indicate that *PsHSP20-6* may be regulated by multiple TFs, participating in diverse transcriptional regulatory stages. Collectively, the *PsHSP20* family likely integrates signals from upstream TFs to form a complex regulatory network, enhancing stress adaptation in the peony.

### 3.7. Regulatory Network and Functional Enrichment of the PsHSP20 Genes

**Figure 7:**
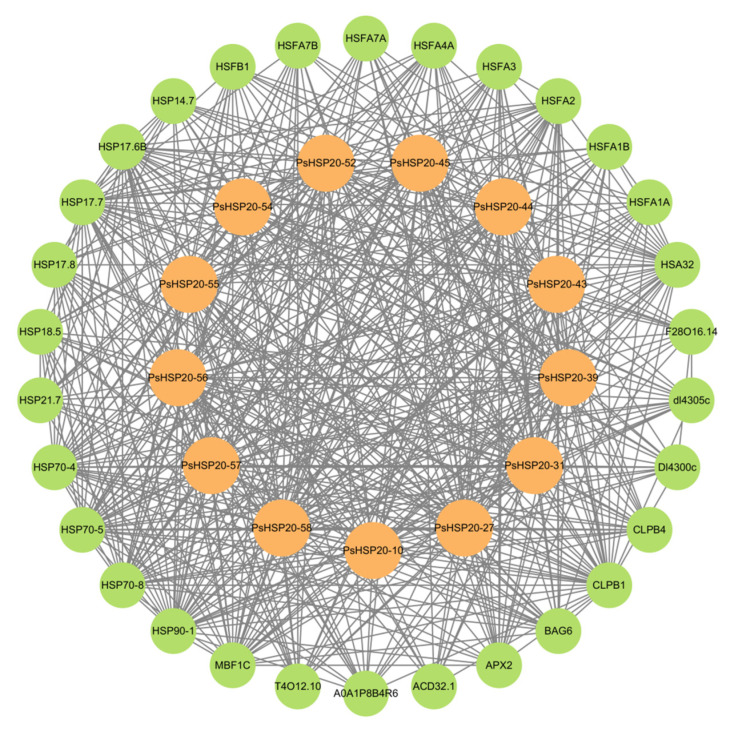
Protein–protein interaction (PPI) network of the *PsHSP20* genes. The network was constructed based on orthologous interactions in *Arabidopsis*. Nodes represent *PsHSP20s* (orange) and their interactors (green), while edges indicate functional associations. Key hub genes (e.g., *PsHSP20-58*) are labeled.

**Figure 8:**
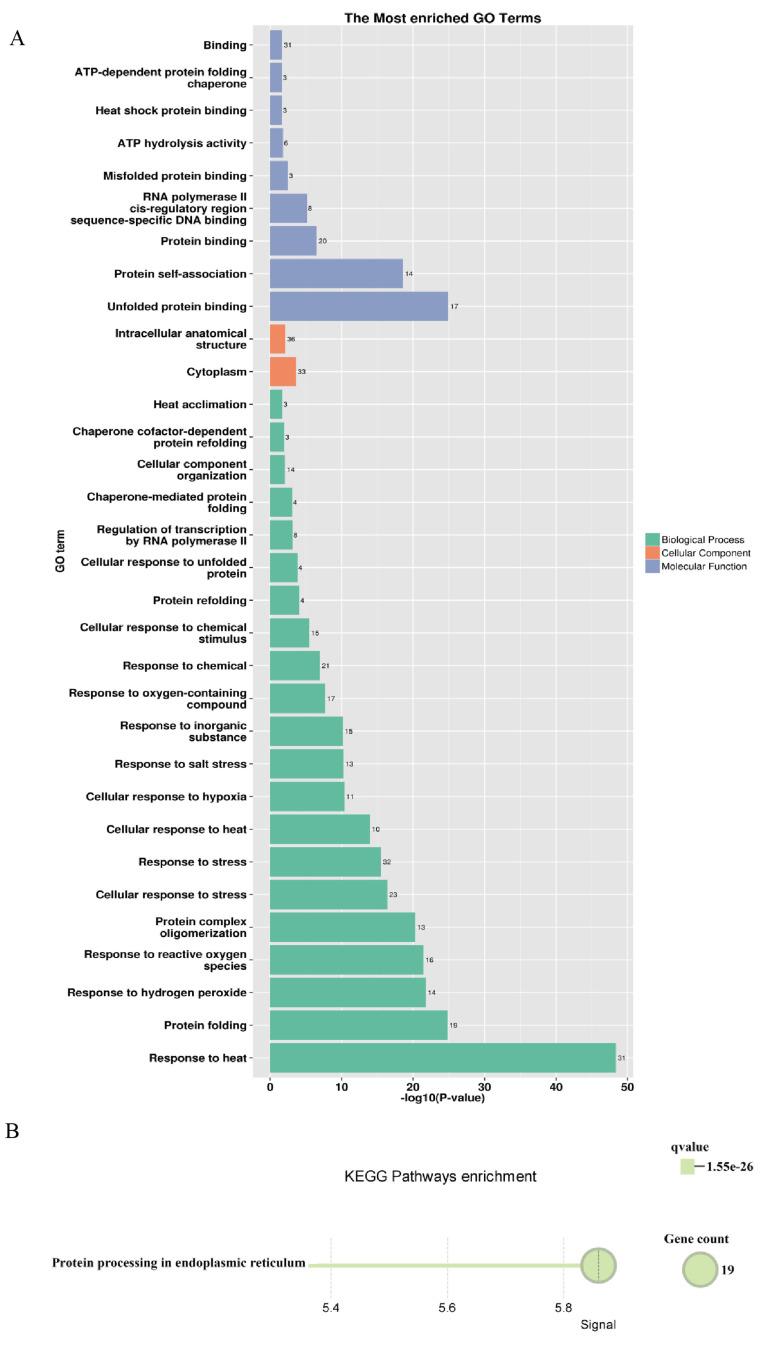
Functional enrichment analysis of the *PsHSP20* interacting genes: (**A**) GO enrichment analysis showing significant terms in the biological processes (e.g., ‘response to heat’), molecular functions (e.g., ‘unfolded protein binding’), and cellular components; (**B**) KEGG pathway enrichment analysis. The top enriched pathway is ‘protein processing in endoplasmic reticulum’.

A regulatory network of the *PsHSP20s* in the peony was constructed based on the synteny analysis with the *Arabidopsis* genome (Figure 7). Among the network components, 13 genes exhibited homology to *Arabidopsis*, and 30 interacting genes were identified, predominantly enriched in the HSF and HSP families, including APX2, BAG6, and CLPB1. This highlights the broad involvement of the *PsHSP20s* in stress responses and developmental regulation in the peony. The hub gene *PsHSP20-58* displayed the highest number of interaction targets (26), implicating it in diverse biological processes, such as abiotic stress, apoptosis, and developmental regulation. Functional redundancy was observed between *PsHSP20-39* and *PsHSP20-45*, suggesting their cooperative regulation of shared biological pathways. Further the GO/KEGG enrichment analyses (Figure 8) revealed significant associations of the interacting genes, with 9 molecular functions, 21 biological processes, and 2 cellular components. The most enriched GO terms included ‘response to heat’, ‘unfolded protein binding’, ‘protein binding’, and ‘reactive oxygen species metabolism’. A KEGG pathway analysis showed that 19 genes were clustered in ‘protein processing in endoplasmic reticulum’, consistent with the GO enrichment results.

### 3.8. Dynamic Expression Profiling of the PsHSP20 Genes Under Heat Stress

**Figure 9:**
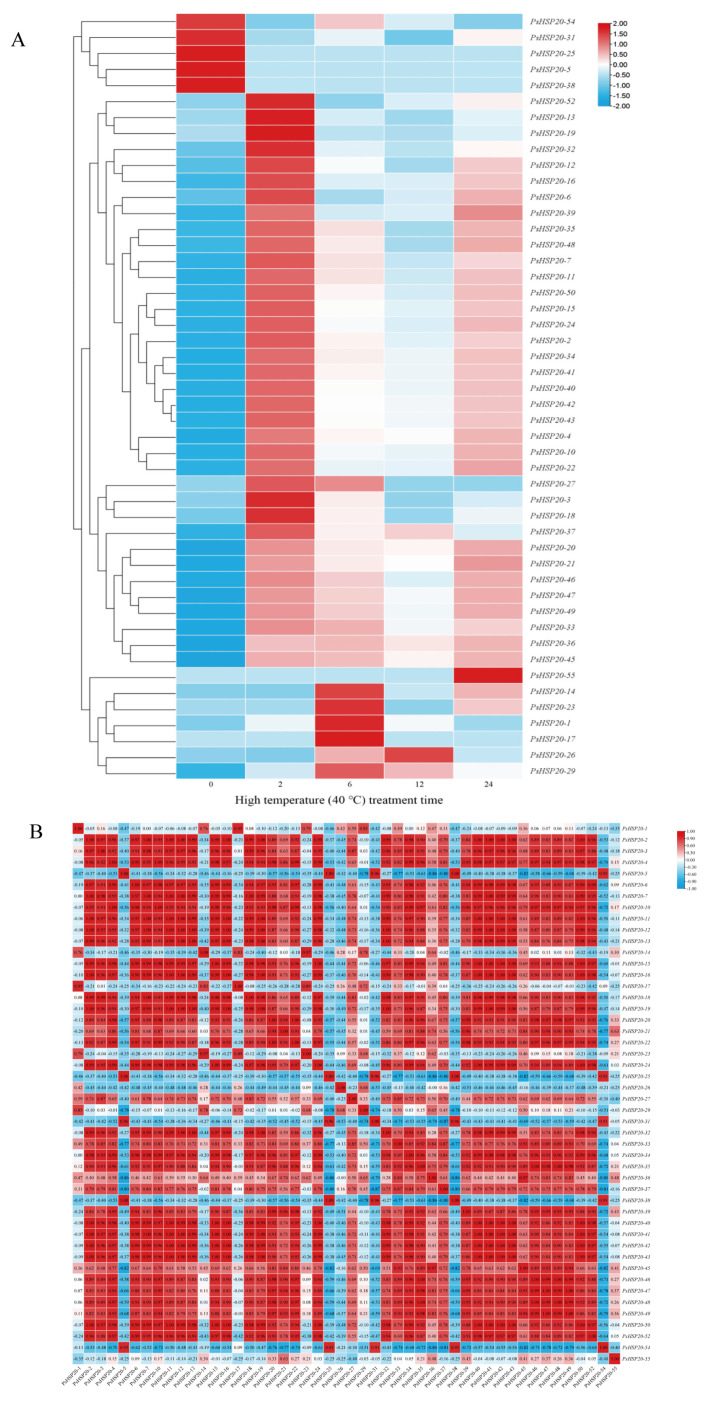
Expression patterns and co-expression analysis of the *PsHSP20* genes under 40 °C heat stress: (**A**) Heatmap of 48 differentially expressed *PsHSP20* genes clustered into two major groups (high- and low-expression). Expression levels are normalized and color-coded (red: upregulation; blue: downregulation); (**B**) Correlation matrix of gene expression profiles. Red squares indicate significant positive correlations (|r| ≧ 0.9).

A hierarchical clustering analysis of the transcriptomic data under heat stress revealed that 48 out of 58 *PsHSP20* genes exhibited significant differential expression (Figure 9A). These genes were categorized into two groups: a constitutively high-expression group (5 genes) and a constitutively low-expression group (43 genes) under non-stress conditions. The high-expression group was further divided into two subtypes: Subtype I (*PsHSP20-54*, *PsHSP20-31*) showed transient downregulation followed by a brief recovery during the early stress phase (0–2 h), while Subtype II (*PsHSP20-25*, *PsHSP20-5, PsHSP20-38*) displayed rapid downregulation and sustained low expression. The low-expression group exhibited six dynamic response patterns: Subtype I (4 genes) peaked at 2 h post-stress before declining; Subtype II (20 genes) oscillated in an ‘up-down-up’ pattern; Subtype III (4 genes) rose rapidly in the early stages followed by a gradual decline; Subtype IV (8 genes) showed phasic fluctuations; Subtype V (*PsHSP20-55*) was transiently activated at 24h; and Subtype VI (6 genes) was specifically induced between 6 and 12 h (Figure 9A). Notably, 24 genes (Subtypes I and II) shared expression peaks synchronized with *PsHSFA3* at 2 h, suggesting direct regulatory control by *PsHSFA3*. A correlation analysis of the expression levels under heat stress identified 369 significant positive correlations (|r| ≧ 0.9) among 48 *PsHSP20* genes, including 11 perfectly co-expressed gene pairs (r = 1.0) with no significant negative correlations observed (Figure 9B). These results demonstrate that the *PsHSP20* family coordinates a synergistic regulatory network to combat heat stress, with functional redundancy likely serving as a critical molecular mechanism underpinning thermotolerance in the peony.

### 3.9. qRT-PCR Analysis of Target Genes of the PsHSP20 Gene Family

**Figure 10:**
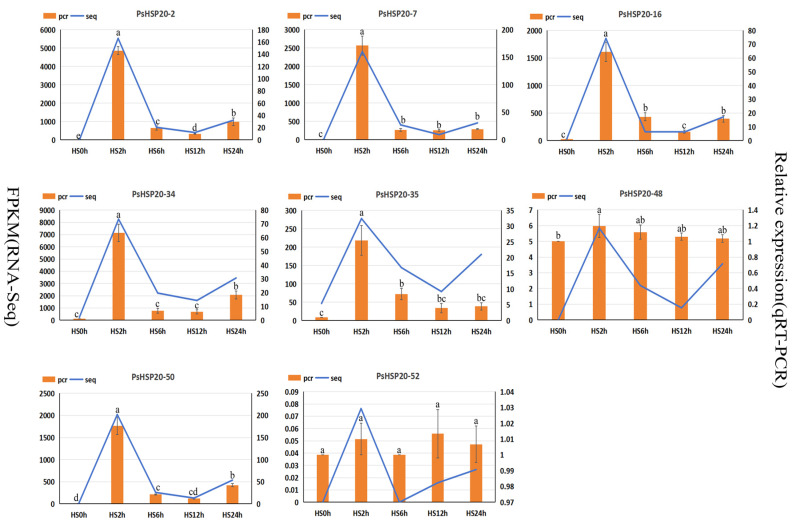
The qRT-PCR analysis of the expression of the downstream target gene *PsHSP20* under high temperature stress at 40 °C. Different letters a and b are used to indicate significant difference (*p* < 0.05); otherwise, the difference is not significant. The data are shown as the mean ± SDs.

To verify the gene expression data, eight candidate genes induced by high temperature were randomly selected according to different subfamilies, and their expression patterns during the high-temperature stress process were analyzed by fluorescence quantitative PCR (Figure 10). The results indicated that, among the eight candidate genes, the expression levels of six genes decreased rapidly after being significantly induced under high-temperature stress for 2 h, and all reached their peak at 2 h. Among them, the expression levels of *PsHSP20-2*, *PsHSP20-16*, *PsHSP20-34*, and *PsHSP20-50* showed a slight increase at 24 h under high-temperature stress. In addition, the expression levels of *PsHSP20-48* and *PsHSP20-52* showed no obvious changing trends under normal and high-temperature stresses. Among them, the expression level of *PsHSP20-52* was not significant in any period. The expression trends of the other genes were consistent with the transcriptome results, verifying the accuracy of the transcriptome data.

## 4. Discussion

*HSP20* proteins, functioning as molecular chaperones, mitigate the irreversible aggregation of denatured proteins to enhance plant stress resilience. Their roles have been extensively characterized in diverse species, including wheat (*Triticum aestivum*) [22], cucumber (*Cucumis sativus* L.) [23], peach (*Prunus persica*) [24], rice (*O. sativa*) [25], rapeseed (*Brassica napus*) [26], and the model plant (*A. thaliana*) [27]. In this study, we identified 58 *PsHSP20* genes in the peony, a gene family size that significantly exceeds those reported in *Arabidopsis* [28,29,30] and *Oryza* [31,32,33], aligning with the expansions observed in *Cucumis melo* (melon) [34]. All 58 *PsHSP20* genes harbor the conserved *HSP20* domain, encoding proteins with molecular weights ranging from 15.19 to 40.88 kDa, consistent with the typical molecular weight range (12–42 kDa) of plant small heat shock proteins.

The majority of the *PsHSP20* proteins were classified as intrinsically unstable, a hallmark of stress-responsive proteins that align with their rapid transcriptional induction under stress conditions [35,36]. Except for *PsHSP20-8* (hydrophilicity coefficient: 0.06), all *PsHSP20s* exhibited hydrophilic properties. Subcellular localization predictions revealed distinct compartmentalization: 49 proteins were localized to the cytoplasm/nucleus, 5 to mitochondria, *PsHSP20-35* and *PsHSP20-56* to peroxisomes, *PsHSP20-45* to the extracellular matrix, and *PsHSP20-44* to chloroplasts. The subcellular localization of *PsHSP20s* dictates their distinct biological functions [37], underscoring their critical roles in diverse cellular compartments of the peony. The amino acid sequence alignment confirmed that all 58 *PsHSP20* genes harbor the conserved ACD (α-crystallin domain), consistent with the findings reported by Yan et al. [37].

The phylogenetic classification revealed 11 subfamilies of the *PsHSP20s*, a structure closely aligned with those of *A. thaliana* (12 subfamilies) [38,39] and *O. sativa* (10 subfamilies) [22]. Notably, the *PsHSP20s* exhibited closer evolutionary affinities to *Arabidopsis* than to *Oryza*, suggesting lineage-specific divergence. Exon–intron architectures, known to drive evolutionary innovation in gene families [32,40], likely contributed to this phylogenetic distinction. The genomic architecture of the *PsHSP20* genes is notably streamlined, with 39.66% (23 genes) lacking introns and 34.48% (20 genes) containing only one intron. This structural simplicity may be evolutionarily advantageous, likely attributed to the preferential retention of the intron-poor or intronless genes in plants to facilitate rapid stress-responsive activation. Such genomic configurations enable swift transcriptional induction under diverse environmental stresses, as minimal intronic content reduces transcriptional latency [27]. As members of the *HSP20* gene family, which are hallmark rapid responders to abiotic stresses [25], *PsHSP20s* are hypothesized to leverage this streamlined architecture for expedited stress perception and signaling, underscoring their pivotal role in the peony’s adaptive plasticity.

Motif1 and Motif2 were the most prevalent motifs across the *PsHSP20* sequences, likely representing core conserved domains of this gene family, with Motif2 exhibiting the highest degree of sequence conservation. Members within the same *PsHSP20* subclade shared nearly identical motif architectures, whereas significant compositional divergence was observed between subclades. These findings indicate structural and functional diversification of the *PsHSP20s* during evolution, further corroborating phylogenetic clustering patterns. Notably, *PsHSP20-17* uniquely harbors both the ACD domain and a PPR2 domain, highlighting functional heterogeneity within the *PsHSP20* family despite their conserved roles, suggesting lineage-specific neofunctionalization.

Fifty-five *PsHSP20* genes were unevenly distributed across all five chromosomes of the peony, with Chr05 harboring the highest gene count (18 genes), suggesting its potential role as a key chromosomal locus for abiotic stress adaptation. While gene duplication events are pivotal drivers of gene family expansion and environmental adaptation in plants [13], no such events were detected within the *PsHSP20* family. A comparative genomic analysis revealed a higher number of orthologous gene pairs between the *PsHSP20s* and the *AtHSP20s* than the *OsHSP20s*, indicating closer evolutionary homology between the *AtHSP20s* and the *PsHSP20s*.

Under stress conditions, promoter-located cis-acting elements associated with stress responses play critical roles in transcriptional regulation [41]. In this study, cis-elements linked to abiotic stress and phytohormone signaling accounted for the majority of those identified in the *PsHSP20* promoters, suggesting that this gene family likely mediates both abiotic stress adaptation and hormonal crosstalk. The combinatorial binding of TFs to these cis-elements fine-tunes spatiotemporal gene expression patterns under varying environmental conditions, ultimately determining the biological functionality of the *PsHSP20s* [40]. The promoter regions of the *PsHSP20* genes are predicted to harbor 24 TF binding motifs, suggesting that this family is regulated by diverse upstream TFs and plays a critical role in the peony’s adaptation to environmental stresses. This aligns with the canonical role of *HSP20s* as downstream effectors within regulatory networks, governed by intricate transcriptional cascades. Notably, binding motifs for key stress-responsive TFs, including HSF [41], MYB [42], and AP2/ERF [12], were identified, implicating the *PsHSP20s* in thermotolerance regulation. Strikingly, 16 *PsHSP20* genes contained predicted HSF-binding sites, further supporting their centrality in heat stress signaling pathways.

A protein–protein interaction (PPI) analysis via the STRING database predicted functional associations between 30 genes and 13 *PsHSP20s*, with most interactors belonging to the *HSF* (heat shock factor) and the *HSP* (heat shock protein) families. In plants, the *HSFs* regulate heat stress responses by binding to the heat shock elements (*HSEs*) in the promoter regions of the *HSP* gene [43]. This suggests that the *PsHSFs* and *PsHSP20s* in the peony may coordinate through reciprocal recognition of the *HSEs* in their promoters. While a PPI network analysis predicts interactions between key *PsHSP20s* and components of the heat shock response machine, definitive validation of these protein–protein interactions through experimental approaches, such as yeast two-hybrid (Y2H), co-immunoprecipitation (Co-IP), or bimolecular fluorescence complementation (BiFC), is essential to confirm their role in the rapid assembly of protective complexes during heat stress [6,44]. Future studies focusing on the interactome of core, rapidly induced *PsHSP20s* (e.g., PsHSP20-54, PsHSP20-31) will be crucial to bridge the gap between rapid transcriptional induction and the establishment of functional thermotolerance.

The GO enrichment analysis revealed that genes interacting with the *PsHSP20s* were most significantly enriched in the ‘response to heat’ pathway. The KEGG analysis further demonstrated that 19 interacting genes clustered in the ‘protein processing in endoplasmic reticulum’ pathway, which governs critical post-translational mechanisms, including protein folding, modification, and trafficking. Proteins must undergo proper folding to be recognized by their biological targets and fulfill their functions. This aligns with the canonical role of *HSP* genes as downstream effectors in regulatory networks, where their activity is precisely modulated by upstream transcription factors. A transcriptomic analysis of the peony under heat stress revealed differential expression levels of 48 out of 58 *PsHSP20* family genes across various treatment time points, indicating their dynamic regulatory roles in heat stress adaptation.

Under heat stress, approximately 89.58% of the *PsHSP20s* genes exhibited upregulated expression, with the majority showing rapid transcriptional activation within 2 h of exposure, underscoring their high sensitivity to thermal stress, these results are consistent with previous reports [16,45]. Notably, among the constitutively low-expressed genes under non-stress conditions, 24 genes from Subtypes I and II displayed expression patterns highly synchronized with *PsHSFA3*, suggesting that their heat-induced expression may be directly regulated by *PsHSFA3*, a key transcription factor in heat stress signaling. To solidify these bioinformatic predictions and experimental observations, and to advance our understanding of PsHSP20 functions, future investigations should prioritize the functional characterization (via gene knockout/knockdown or overexpression) of key PsHSP20 candidates, particularly those showing strong and rapid induction. The integration of more detailed transcriptomic time-course analyses, employing heat-map visualizations, to comprehensively map the expression dynamics across the entire gene family under varying heat stress intensities and durations should also be investigated.

## 5. Conclusions

We conducted a genome-wide identification and bioinformatics analysis of the *PsHSP20* gene family in the peony. A total of 58 *PsHSP20* genes were identified from the peony genome, and the *PsHSP20* proteins are localized to diverse subcellular compartments. Phylogenetically, the *PsHSP20* family is divided into 11 subfamilies, all harboring the conserved *HSP20* domain (ACD, α-crystallin domain). Fifty-five *PsHSP20* genes are unevenly distributed across all five chromosomes of the peony, with no intra-species gene duplication events detected. A comparative genomic analysis revealed a higher number of orthologous gene pairs between the *PsHSP20s* and the *A. thaliana* HSP20s (*AtHSP20s*) than the *O. sativa* HSP20s (*OsHSP20s*), indicating closer evolutionary homology between the *AtHSP20s* and the *PsHSP20s*. Promoter regions of the *PsHSP20* genes are enriched with diverse cis-regulatory elements associated with phytohormone signaling and abiotic stress responses, along with predicted binding sites for 24 transcription factor families (e.g., HSF, MYB, AP2/ERF). Among these, 16 genes were predicted to harbor *HSF* transcription factor binding sites. A protein–protein interaction (PPI) network analysis identified 30 genes interacting with 13 *PsHSP20s*, predominantly enriched in the ‘response to heat’ GO term and the ‘protein processing in endoplasmic reticulum’ KEGG pathway. Importantly, the qRT-PCR validation of eight selected PsHSP20s genes confirmed the rapid induction and expression trends observed in the transcriptome data for most targets, with six genes peaking at 2 h post-heat stress. This experimental validation strengthens confidence in the transcriptome-derived expression patterns of the PsHSP20 family during heat stress. This research offers valuable reference data for exploring the regulation of the *PsHSP20* family in the peony’s response to high-temperature stress, as well as its role in growth and development in the peony.

## Data Availability

The data used in this study were downloaded from the NCBI website, the CNSA website, and the Gramene database, and their links were marked in both the materials and methods.

## Supplementary Materials

The following supporting information can be downloaded at: https://www.mdpi.com/article/10.3390/genes16070742/s1, Figure S1. Number of predicted cis-elements in the promoter of *PsHSP20s* gene family in *Paeonia suffruticosa*; Figure S2. Number of predicted transcription factor binding sites in the promoter of *PsHSP20s* gene family in *Paeonia suffruticosa*; Table S1. Primer sequence of qRT-PCR gene; Table S2. Physicochemical properties of *PsHSP20s* gene family in peony; Table S3. Prediction of secondary structure of *PsHSP20s* gene family protein in peony(%); Table S4 Chromosome localization information of *PsHSP20s* gene in peony.

## Abbreviations

The following abbreviations are used in this manuscript:

ACD	α-crystallin Domain
CDD	Conserved Domain Database
CDS	Coding Sequence
GO	Gene Ontology
HMM	Hidden Markov Model
HSE	Heat Shock Elements
HSP	Heat Shock Protein
KEGG	Kyoto Encyclopedia of Genes and Genomes
LCA	Luciferase Complementarity Assays
PPI	Protein–Protein Interaction
TF	Transcription Factor

## References

[1] Tong N., Shu Q., Wang B., Peng L., Liu Z. (2023). Histology, Physiology, and Transcriptomic and Metabolomic Profiling Reveal the Developmental Dynamics of Annual Shoots in Tree Peonies (*Paeonia suffruticosa* Andr.). Hortic. Res..

[2] Zhao D., Xia X., Su J., Wei M., Wu Y., Tao J. (2019). Overexpression of Herbaceous Peony HSP70 Confers High Temperature Tolerance. BMC Genom..

[3] Wang Q., Zhou L., Yuan M., Peng F., Zhu X., Wang Y. (2024). Genome-Wide Identification of NAC Gene Family Members of Tree Peony (*Paeonia suffruticosa* Andrews) and Their Expression under Heat and Waterlogging Stress. Int. J. Mol. Sci..

[4] Li Z., Ji W., Hong E., Fan Z., Lin B., Xia X., Chen X., Zhu X. (2023). Study on Heat Resistance of Peony Using Photosynthetic Indexes and Rapid Fluorescence Kinetics. Horticulturae.

[5] Ma J., Wang Q., Wei L.-L., Zhao Y., Zhang G.-Z., Wang J., Gu C.-H. (2022). Responses of the Tree Peony (*Paeonia suffruticosa*, Paeoniaceae) Cultivar “Yu Hong” to Heat Stress Revealed by iTRAQ-Based Quantitative Proteomics. Proteome Sci..

[6] Bu W., Huang Y., Chen L., Zhang M., Luo X., Zheng T., Shao F., Lei W., Xing W., Yang X. (2025). Transcriptome Analysis of Tree Peony under High Temperature Treatment and Functional Verification of PsDREB2A Gene. Plant Physiol. Biochem..

[7] Jagadish S.V.K., Way D.A., Sharkey T.D. (2021). Plant Heat Stress: Concepts Directing Future Research. Plant Cell Environ..

[8] Ohama N., Sato H., Shinozaki K., Yamaguchi-Shinozaki K. (2017). Transcriptional Regulatory Network of Plant Heat Stress Response. Trends Plant Sci..

[9] Wang L., Hou J., Wang J., Zhu Z., Zhang W., Zhang X., Shen H., Wang X. (2020). Regulatory Roles of HSPA6 in *Actinidia chinensis* Planch. Root Extract (acRoots)-Inhibited Lung Cancer Proliferation. Clin. Transl. Med..

[10] Yang X., Huang Y., Yao Y., Bu W., Zhang M., Zheng T., Luo X., Wang Z., Lei W., Tian J. (2024). Mining Heat-Resistant Key Genes of Peony Based on Weighted Gene Co-Expression Network Analysis. Genes.

[11] Huang L.-Z., Zhou M., Ding Y.-F., Zhu C. (2022). Gene Networks Involved in Plant Heat Stress Response and Tolerance. Int. J. Mol. Sci..

[12] Yuan J., Jiang S., Jian J., Liu M., Yue Z., Xu J., Li J., Xu C., Lin L., Jing Y. (2022). Genomic Basis of the Giga-Chromosomes and Giga-Genome of Tree Peony *Paeonia ostii*. Nat. Commun..

[13] Wang Q., Li B., Qiu Z., Lu Z., Hang Z., Wu F., Chen X., Zhu X. (2024). Genome-Wide Identification of MYC Transcription Factors and Their Potential Functions in the Growth and Development Regulation of Tree Peony (*Paeonia suffruticosa*). Plants.

[14] Wang Q., Sun W., Duan Y., Xu Y., Wang H., Hao J., Han Y., Liu C. (2024). Genome-Wide Identification and Expression Analysis of HSP70 Gene Family Under High-Temperature Stress in Lettuce (*Lactuca sativa* L.). Int. J. Mol. Sci..

[15] Peng L., Song W., Tan W., Liu Z., Wang X., Li Y., Shu Q. (2023). Integration of Genome-Wide Identification, Transcriptome and Association Analysis of *HSP20* Gene Family to Revealing Genetic Basis of Floral Organ Number-Related Traits in Tree Peony. Ornam. Plant Res..

[16] Hu Y., Zhang T., Liu Y., Li Y., Wang M., Zhu B., Liao D., Yun T., Huang W., Zhang W. (2021). Pumpkin (*Cucurbita moschata*) HSP20 Gene Family Identification and Expression Under Heat Stress. Front. Genet..

[17] Cho E.K., Choi Y.J. (2009). A Nuclear-Localized HSP70 Confers Thermoprotective Activity and Drought-Stress Tolerance on Plants. Biotechnol. Lett..

[18] Sato H., Mizoi J., Shinozaki K., Yamaguchi-Shinozaki K. (2024). Complex Plant Responses to Drought and Heat Stress under Climate Change. Plant J..

[19] Huang B., Xu C. (2008). Identification and Characterization of Proteins Associated with Plant Tolerance to Heat Stress. J. Integr. Plant Biol..

[20] Chen C., Chen H., Zhang Y., Thomas H.R., Xia R. (2020). TBtools: An Integrative Toolkit Developed for Interactive Analyses of Big Biological Data. Mol. Plant.

[21] Bailey T.L., Boden M., Buske F.A., Frith M., Grant C.E., Clementi L., Ren J., Li W.W., Noble W.S. (2009). MEME SUITE: Tools for Motif Discovery and Searching. Nucleic Acids Res..

[22] Guo L.-M., Li J., He J., Liu H., Zhang H.-M. (2020). A Class I Cytosolic HSP20 of Rice Enhances Heat and Salt Tolerance in Different Organisms. Sci. Rep..

[23] Huang J., Hai Z., Wang R., Yu Y., Chen X., Liang W., Wang H. (2022). Genome-Wide Analysis of HSP20 Gene Family and Expression Patterns under Heat Stress in Cucumber (*Cucumis sativus* L.). Front. Plant Sci..

[24] Lian X., Wang Q., Li T., Gao H., Li H., Zheng X., Wang X., Zhang H., Cheng J., Wang W. (2022). Phylogenetic and Transcriptional Analyses of the HSP20 Gene Family in Peach Revealed That PpHSP20-32 Is Involved in Plant Height and Heat Tolerance. Int. J. Mol. Sci..

[25] Sarkar N.K., Kim Y.-K., Grover A. (2009). Rice sHsp Genes: Genomic Organization and Expression Profiling under Stress and Development. BMC Genom..

[26] Wang X., Yan B., Shi M., Zhou W., Zekria D., Wang H., Kai G. (2016). Overexpression of a *Brassica campestris* HSP70 in Tobacco Confers Enhanced Tolerance to Heat Stress. Protoplasma.

[27] Chung B.Y.W., Simons C., Firth A.E., Brown C.M., Hellens R.P. (2006). Effect of 5′UTR Introns on Gene Expression in *Arabidopsis thaliana*. BMC Genom..

[28] Lämke J., Brzezinka K., Bäurle I. (2016). HSFA2 Orchestrates Transcriptional Dynamics after Heat Stress in *Arabidopsis thaliana*. Transcription.

[29] Tsai W.-A., Sung P.-H., Kuo Y.-W., Chen M.-C., Jeng S.-T., Lin J.-S. (2023). Involvement of microRNA164 in Responses to Heat Stress in Arabidopsis. Plant Sci..

[30] Wang J., Gao X., Dong J., Tian X., Wang J., Palta J.A., Xu S., Fang Y., Wang Z. (2020). Over-Expression of the Heat-Responsive Wheat Gene TaHSP23.9 in Transgenic Arabidopsis Conferred Tolerance to Heat and Salt Stress. Front. Plant Sci..

[31] Zhang H., Zhou J.-F., Kan Y., Shan J.-X., Ye W.-W., Dong N.-Q., Guo T., Xiang Y.-H., Yang Y.-B., Li Y.-C. (2022). A Genetic Module at One Locus in Rice Protects Chloroplasts to Enhance Thermotolerance. Science.

[32] Chang Y., Fang Y., Liu J., Ye T., Li X., Tu H., Ye Y., Wang Y., Xiong L. (2024). Stress-Induced Nuclear Translocation of ONAC023 Improves Drought and Heat Tolerance Through Multiple Processes in Rice. Nat. Commun..

[33] Ren Y., Huang Z., Jiang H., Wang Z., Wu F., Xiong Y., Yao J. (2021). A Heat Stress Responsive NAC Transcription Factor Heterodimer Plays Key Roles in Rice Grain Filling. J. Exp. Bot..

[34] Piñero M.C., Otálora G., Collado J., López-Marín J., Del Amor F.M. (2021). Foliar Application of Putrescine before a Short-Term Heat Stress Improves the Quality of Melon Fruits (*Cucumis melo* L.). J. Sci. Food Agric..

[35] Hua Y., Liu Q., Zhai Y., Zhao L., Zhu J., Zhang X., Jia Q., Liang Z., Wang D. (2023). Genome-Wide Analysis of the HSP20 Gene Family and Its Response to Heat and Drought Stress in Coix (*Coix lacryma-jobi* L.). BMC Genom..

[36] Zhang C., Zhang Y., Su Z., Shen Z., Song H., Cai Z., Xu J., Guo L., Zhang Y., Guo S. (2023). Integrated Analysis of HSP20 Genes in the Developing Flesh of Peach: Identification, Expression Profiling, and Subcellular Localization. BMC Plant Biol..

[37] Yan H., Du M., Ding J., Song D., Ma W., Li Y. (2024). Pan-Genome-Wide Investigation and Co-Expression Network Analysis of HSP20 Gene Family in Maize. Int. J. Mol. Sci..

[38] Zhong L., Shi Y., Xu S., Xie S., Huang X., Li Y., Qu C., Liu J., Liao J., Huang Y. (2024). Heterologous Overexpression of Heat Shock Protein 20 Genes of Different Species of Yellow Camellia in *Arabidopsis thaliana* Reveals Their Roles in High Calcium Resistance. BMC Plant Biol..

[39] Qi X., Di Z., Li Y., Zhang Z., Guo M., Tong B., Lu Y., Zhang Y., Zheng J. (2022). Genome-Wide Identification and Expression Profiling of Heat Shock Protein 20 Gene Family in *Sorbus pohuashanensis* (Hance) Hedl under Abiotic Stress. Genes.

[40] Ji H., Liu J., Chen Y., Yu X., Luo C., Sang L., Zhou J., Liao H. (2024). Bioinformatic Analysis of Codon Usage Bias of HSP20 Genes in *Four cruciferous* Species. Plants.

[41] Kan Y., Mu X.-R., Gao J., Lin H.-X., Lin Y. (2023). The Molecular Basis of Heat Stress Responses in Plants. Mol. Plant.

[42] Wu Y., Li X., Zhang J., Zhao H., Tan S., Xu W., Pan J., Yang F., Pi E. (2022). ERF Subfamily Transcription Factors and Their Function in Plant Responses to Abiotic Stresses. Front. Plant Sci..

[43] Cui F., Taier G., Wang X., Wang K. (2021). Genome-Wide Analysis of the HSP20 Gene Family and Expression Patterns of HSP20 Genes in Response to Abiotic Stresses in *Cynodon transvaalensis*. Front. Genet..

[44] Wang X., Tan N.W.K., Chung F.Y., Yamaguchi N., Gan E.-S., Ito T. (2023). Transcriptional Regulators of Plant Adaptation to Heat Stress. Int. J. Mol. Sci..

[45] Li N., Jiang M., Li P., Li X. (2021). Identification, Expression, and Functional Analysis of Hsf and Hsp20 Gene Families in Brachypodium Distachyon under Heat Stress. PeerJ.

