# Supplementary material for: Identification and Bioinformatics Analysis of the HSP20 Family in the Peony
Source: Genes (Basel). 2025 Jun 26;16(7):742. doi: 10.3390/genes16070742 (PMC12294252; doi:10.3390/genes16070742)
Supplement: Supplementary file 1 [file genes-16-00742-s001.zip › genes-3686538-supplementary.pdf]

Supplementary Figure S1. Number of predicted cis-elements in the promoter of *PsHSP20s* gene family in *Paeonia suffruticosa*

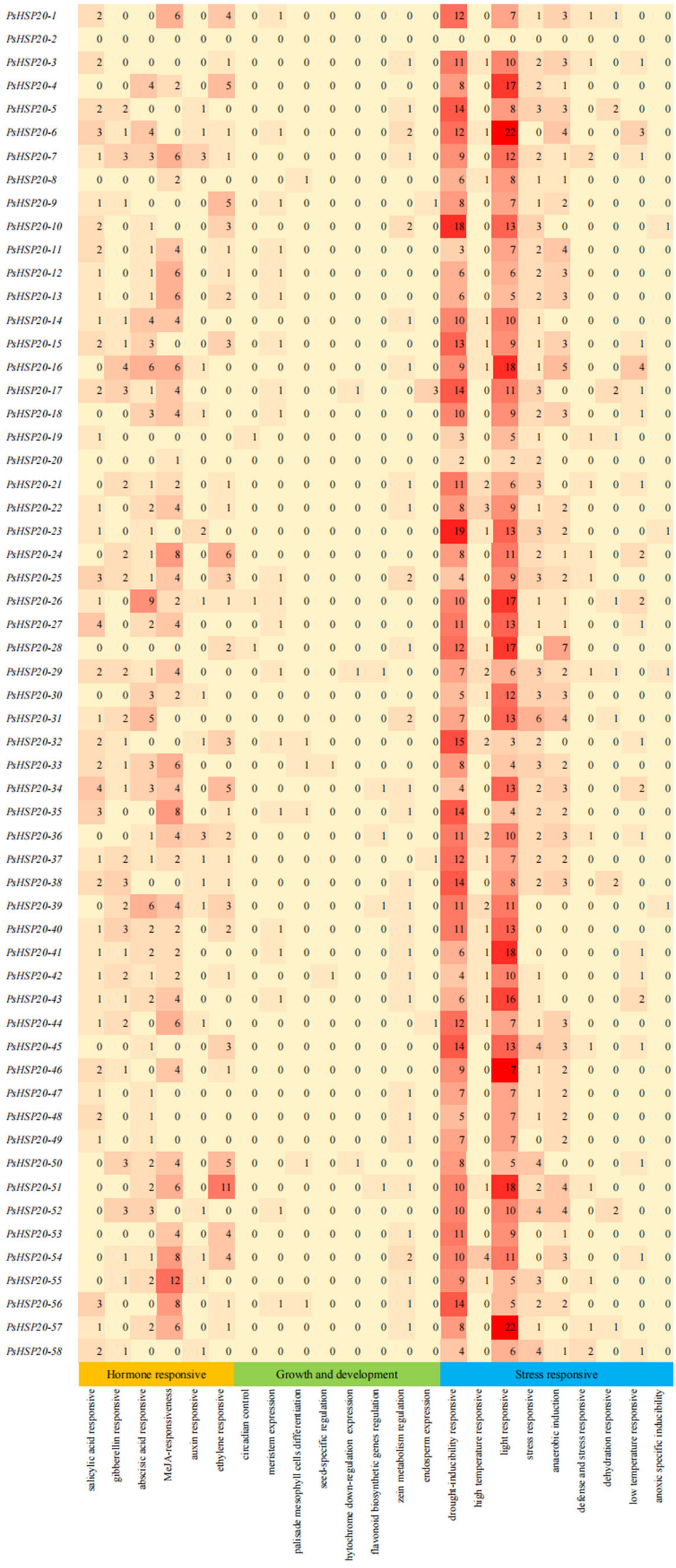

Supplementary Figure S2. Number of predicted transcription factor binding sites in the promoter of *PsHSP20s* gene family in *Paeonia suffruticosa*

	Dof	BBR-BPC	WRKY	TALE	HD-ZIP	MIKC_MADS	HSF	C2H2	MYB	bZIP	Trihelix	ERF	MYB_related	GATA	G2-like	TCP	WOX	RAV	B3	NAC	SBP	bHLH	AP2	C3H
PsHSP20-1	15	0	0	0	0	0	0	0	3	0	0	0	0	0	0	0	1	0	0	0	0	0	0	0
PsHSP20-2	8	0	0	0	0	0	5	0	3	0	0	0	0	0	0	0	0	0	0	0	0	0	0	1
PsHSP20-3	6	0	43	0	3	0	0	1	0	0	1	2	0	1	0	0	0	0	0	0	1	0	0	0
PsHSP20-4	2	0	0	0	3	0	0	2	0	0	0	0	0	0	0	0	0	0	0	0	0	0	0	0
PsHSP20-5	39	0	0	0	1	0	4	0	0	0	1	0	0	0	0	1	0	0	0	0	0	0	0	0
PsHSP20-6	6	0	0	0	2	0	0	0	2	1	2	0	1	0	1	2	0	0	0	1	0	0	0	0
PsHSP20-7	12	0	0	0	0	1	5	1	0	0	1	2	1	0	0	0	0	0	0	0	0	0	0	0
PsHSP20-8	1	0	23	0	0	2	0	0	0	0	0	0	0	0	0	0	0	0	0	2	0	0	0	0
PsHSP20-9	0	0	0	0	2	0	0	0	0	0	0	0	0	0	0	0	0	0	0	0	0	0	0	0
PsHSP20-10	12	0	0	0	1	0	0	0	0	0	1	0	0	0	0	0	0	0	0	0	0	0	0	0
PsHSP20-11	0	33	0	13	0	1	0	2	0	0	0	0	0	0	0	0	0	0	0	0	0	0	0	0
PsHSP20-12	0	0	0	0	0	0	0	0	0	0	0	0	0	0	0	0	0	0	0	0	1	0	0	0
PsHSP20-13	2	0	0	0	0	0	0	0	0	0	0	0	0	0	0	0	0	0	0	0	1	0	0	0
PsHSP20-14	30	0	13	0	0	1	0	1	1	0	1	2	0	0	0	0	0	0	1	0	0	0	0	0
PsHSP20-15	1	0	0	0	0	1	0	0	0	0	0	0	1	0	0	0	0	0	0	0	0	0	0	0
PsHSP20-16	0	0	0	0	0	0	2	2	0	0	0	0	0	0	0	0	0	0	0	0	0	0	0	0
PsHSP20-17	2	0	0	0	0	0	0	0	0	0	0	0	0	0	1	0	0	0	0	0	0	0	0	0
PsHSP20-18	3	0	0	0	0	0	0	1	0	2	0	0	1	0	0	0	0	0	0	0	0	0	0	0
PsHSP20-19	0	0	6	0	2	0	0	0	4	0	0	0	0	0	0	0	0	1	1	0	0	0	0	0
PsHSP20-20	0	0	0	0	0	0	1	0	1	0	0	0	0	0	0	0	0	0	0	0	0	0	1	0
PsHSP20-21	0	0	0	0	0	1	1	0	0	0	0	0	0	0	0	0	0	0	0	0	0	0	1	0
PsHSP20-22	0	0	39	0	0	0	0	0	0	0	0	0	0	0	0	0	0	0	0	0	0	0	0	0
PsHSP20-23	9	0	0	0	0	0	0	0	1	0	0	0	0	0	2	0	0	0	0	0	0	0	0	0
PsHSP20-24	7	0	2	0	0	1	0	1	1	0	0	0	0	0	0	0	0	0	0	0	0	0	0	0
PsHSP20-25	2	13	0	5	0	0	0	0	0	0	0	0	2	0	1	0	0	0	0	0	0	0	0	0
PsHSP20-26	5	0	1	0	2	0	0	0	0	5	1	0	0	0	0	0	0	0	0	0	0	0	0	0
PsHSP20-27	4	0	0	0	0	0	0	1	0	0	0	0	0	0	0	0	0	0	0	0	0	0	0	0
PsHSP20-28	0	17	0	4	8	0	0	0	0	6	0	0	0	0	0	0	0	0	1	0	0	0	0	0
PsHSP20-29	4	0	0	0	0	0	0	1	0	0	0	0	0	0	0	0	0	0	0	0	0	0	0	0
PsHSP20-30	5	0	1	0	0	0	0	0	2	0	0	1	0	0	0	0	0	0	0	0	0	2	0	0
PsHSP20-31	0	0	1	0	0	0	0	0	0	0	0	0	0	0	0	0	0	0	0	0	0	0	0	0
PsHSP20-32	4	0	0	0	1	0	0	1	1	0	0	0	0	0	0	0	0	0	0	0	0	0	0	0
PsHSP20-33	1	0	0	0	3	12	0	0	0	0	0	0	0	0	0	0	1	0	1	0	0	0	0	0
PsHSP20-34	12	0	0	0	2	2	1	3	0	0	1	0	0	0	0	0	0	0	0	0	0	0	0	0
PsHSP20-35	1	0	0	0	0	0	3	0	0	0	0	0	0	0	0	0	1	0	0	0	0	0	0	0
PsHSP20-36	9	0	0	0	0	0	3	0	0	0	0	0	0	0	2	0	0	0	0	0	0	0	0	1
PsHSP20-37	36	0	0	0	0	1	0	0	0	0	0	0	0	0	0	0	0	0	0	0	0	0	0	0
PsHSP20-38	45	0	0	0	1	0	4	0	0	0	1	0	0	0	0	1	0	1	0	0	0	0	0	0
PsHSP20-39	25	0	0	0	0	2	0	3	0	2	1	0	0	0	0	0	0	0	0	0	0	0	0	0
PsHSP20-40	27	0	0	0	1	4	0	3	0	0	0	0	1	0	0	0	0	0	0	0	0	0	0	0
PsHSP20-41	27	0	0	0	1	0	0	0	0	0	0	0	1	0	0	0	0	0	0	0	0	0	0	0
PsHSP20-42	15	0	0	0	1	0	0	0	0	0	1	0	0	0	0	0	0	0	0	0	0	0	0	0
PsHSP20-43	3	0	0	0	1	1	0	0	0	0	0	0	1	0	0	0	0	0	0	0	0	0	0	0
PsHSP20-44	0	0	0	0	0	1	0	0	0	0	0	0	0	0	0	0	0	0	0	0	0	0	0	0
PsHSP20-45	3	0	0	0	3	1	0	0	0	0	0	0	0	0	0	0	0	0	0	0	0	0	0	0
PsHSP20-46	43	0	0	0	1	0	0	0	0	0	1	0	0	0	0	0	0	0	0	0	0	0	0	0
PsHSP20-47	27	19	0	6	1	0	2	0	0	0	1	0	0	0	1	0	0	1	0	0	0	0	0	0
PsHSP20-48	37	41	0	17	1	0	2	0	0	0	1	0	0	0	0	0	0	0	0	0	0	0	0	0
PsHSP20-49	31	21	0	7	1	0	2	0	0	0	0	0	0	0	1	0	0	0	0	0	0	0	0	0
PsHSP20-50	96	0	0	0	0	1	2	0	0	0	0	0	0	0	0	0	0	0	0	0	0	0	0	0
PsHSP20-51	9	0	0	0	0	0	0	0	2	0	0	0	0	0	0	0	0	0	0	0	0	0	0	0
PsHSP20-52	1	0	1	0	3	0	2	4	0	0	0	3	0	0	0	0	0	0	0	0	0	0	0	0
PsHSP20-53	5	0	0	0	0	3	0	0	4	0	0	0	0	0	0	0	0	0	0	0	0	0	0	0
PsHSP20-54	2	73	0	14	0	0	0	0	0	0	1	2	1	0	0	0	0	0	0	0	0	0	0	0
PsHSP20-55	2	0	0	0	0	1	0	1	0	5	0	1	2	9	0	0	0	0	0	0	0	0	0	0
PsHSP20-56	1	0	0	0	0	0	2	0	0	0	0	0	0	0	0	0	1	0	0	0	0	0	0	0
PsHSP20-57	0	0	0	0	0	3	0	0	0	0	0	0	0	0	0	1	0	1	0	0	0	0	0	0
PsHSP20-58	4	0	0	0	0	4	0	1	0	0	0	1	0	0	1	0	0	0	0	0	0	0	0	0

Supplementary Table S1. Primer sequence of qRT-PCR gene

Gene	Forward primer	Reverse primer
PsActin	CGAATCTTGTCTTGACCCCC	ATTGTCACCACCATCCCTACC
PsHSP20-2	CTGCCTCAAATCTCGTCTCCAAG	GTCATCGCCGTAGTTGCTCAC
PsHSP20-7	TGCCAAGACACTACAACAACTGATG	CCCTATTGTATCCGCCTCCTTCC
PsHSP20-16	GGAGAATGCCAACACCGACAAG	GCAACCTTCACCTCAACAGTCTTAG
PsHSP20-34	AGAGGAAGCGGGAGGAGGAG	ATATCTTGTCGGTGTTGGCATTCTC
PsHSP20-35	ATCGGTTGGAAAGGAGACTGTTTG	CCTGAGCCTTAATCTGGTCTAACTTC
PsHSP20-48	AAGAAGAGGAAGAGAAGAACGACAAG	TCCATACTAGCCTTCACCTCATCC
PsHSP20-50	AGAAGAAGTGAAGGTCGAAGTTGAAG	ACTTGCCGCTGCTCCTCTC
PsHSP20-52	TTGCCTCTGCCGCCATC	TGTGTTCAATCCAGACCTTCAAGTC

Supplementary Table S2. Physicochemical properties of PsHSP20s gene family in peony

Gene	CDS length	Number of amino acids	Molecular weight	Isoelectric point	Instability index	Fat solubility coefficient	Hydrophilicity coefficient	Subcellular localization
PsHSP20-1	480	159	18.54	4.93	60.67	66.1	-0.982	Cytosol
PsHSP20-2	630	209	23.31	5.3	47.48	81.15	-0.53	Mitochondrion
PsHSP20-3	768	255	28.44	6.05	45.54	80.59	-0.502	Nucleus
PsHSP20-4	405	134	15.19	8.76	53.56	70.52	-0.794	Cytosol
PsHSP20-5	435	144	16.19	8.78	44.45	81.11	-0.454	Cytosol
PsHSP20-6	444	147	16.27	7.7	43.45	86.19	-0.382	Nucleus
PsHSP20-7	693	230	25.94	9.06	37.92	66.96	-0.685	Mitochondrion
PsHSP20-8	660	219	24.75	8.44	35.51	109.82	0.06	Cytosol
PsHSP20	477	158	18.47	6.2	64.43	70.95	-0.651	Cytosol

-9								
PsHSP20-10	438	145	16.76	9.26	66.75	70.48	-0.906	Cytosol
PsHSP20-11	489	162	18.36	5.4	59.85	66.79	-0.702	Cytosol
PsHSP20-12	486	161	18.21	5.4	60.16	67.2	-0.724	Cytosol
PsHSP20-13	486	161	18.24	5.4	60.69	67.2	-0.728	Cytosol
PsHSP20-14	480	159	18.04	5.95	35.34	71.64	-0.686	Cytosol
PsHSP20-15	474	157	17.53	5.97	38.71	76.88	-0.492	Cytosol
PsHSP20-16	471	156	17.50	5.79	37.95	77.37	-0.513	Cytosol
PsHSP20-17	834	277	31.22	9.09	52.01	75.96	-0.497	Cytosol
PsHSP20-18	534	177	19.60	6.43	32.11	71.02	-0.405	Nucleus
PsHSP20-19	852	283	32.09	5.92	46.47	84.35	-0.336	Cytosol
PsHSP20-20	579	192	21.68	5.67	43.96	98.96	-0.401	Cytosol
PsHSP20-21	579	192	21.68	5.88	46.12	95.42	-0.44	Cytosol
PsHSP20-22	573	190	21.38	5.52	43.13	100.53	-0.288	Cytosol
PsHSP20-23	447	148	16.87	9.15	40.6	78.31	-0.471	Cytosol
PsHSP20-24	483	160	18.16	5.58	56.58	71.81	-0.643	Cytosol
PsHSP20-25	408	135	15.34	5.4	38.59	83.04	-0.639	Cytosol
PsHSP20-26	480	159	17.88	5.43	48.71	74.09	-0.69	Cytosol
PsHSP20-27	1081	359	40.88	8.23	37.1	97.41	-0.055	Cytosol
PsHSP20-28	675	224	25.56	9.6	27.18	83.93	-0.631	Cytosol
PsHSP20-29	501	166	18.53	4.85	48.32	71.57	-0.63	Mitochondrion
PsHSP20-30	504	167	18.93	5.32	54.61	85.69	-0.419	Cytosol
PsHSP20	690	229	25.94	5.04	46.92	86.29	-0.556	Cytosol

-31								
PsHSP20-32	510	169	18.99	5.96	29.97	79.53	-0.465	Cytosol
PsHSP20-33	486	161	18.33	6.21	73.04	70.87	-0.754	Cytosol
PsHSP20-34	474	157	17.52	5.97	39.54	76.24	-0.505	Cytosol
PsHSP20-35	426	141	16.00	6.3	47.88	76.67	-0.585	Peroxisome
PsHSP20-36	636	211	24.09	9.42	45.06	62.94	-0.822	Mitochondrion
PsHSP20-37	453	150	17.55	8.48	73.75	72.07	-0.857	Nucleus
PsHSP20-38	435	144	16.19	8.78	44.45	81.11	-0.454	Cytosol
PsHSP20-39	672	223	25.31	7.72	52.12	69.51	-0.821	Mitochondrion
PsHSP20-40	498	165	18.73	6.2	50.26	66.12	-0.698	Cytosol
PsHSP20-41	510	169	19.11	5.86	56.19	50.18	-0.957	Cytosol
PsHSP20-42	498	165	18.73	6.2	50.26	66.12	-0.698	Cytosol
PsHSP20-43	498	165	18.73	6.2	50.26	66.12	-0.698	Cytosol
PsHSP20-44	438	145	16.36	9.74	73.29	81.31	-0.629	Chloroplast
PsHSP20-45	714	237	26.27	7.58	52.22	75.65	-0.523	Extracellular matrix
PsHSP20-46	483	160	18.07	5.84	56.44	74.88	-0.61	Cytosol
PsHSP20-47	483	160	18.12	5.58	55.42	73.06	-0.661	Cytosol
PsHSP20-48	507	168	19.12	5.88	57.87	77.68	-0.549	Cytosol
PsHSP20-49	483	160	18.12	5.58	55.42	73.06	-0.661	Cytosol
PsHSP20-50	483	160	18.17	5.58	62.76	73.06	-0.665	Cytosol
PsHSP20-51	447	148	17.01	6.64	57.9	76.35	-0.612	Cytosol
PsHSP20-52	678	225	25.85	6.17	28.41	90.93	-0.564	Cytosol

PsHSP20-53	408	135	15.60	5.58	44.11	85.85	-0.557	Cytosol
PsHSP20-54	414	137	15.65	4.94	50.72	84.74	-0.302	Cytosol
PsHSP20-55	603	200	22.88	6.35	58.23	83.75	-0.402	Cytosol
PsHSP20-56	426	141	16.00	6.3	50.94	76.67	-0.574	Peroxisome
PsHSP20-57	531	176	19.90	8.87	49.28	78.64	-0.518	Nucleus
PsHSP20-58	420	139	15.96	6.61	30.79	81.22	-0.459	Cytosol

Supplementary Table S3. Prediction of secondary structure of PsHSP20s gene family protein in peony(%)

Gene	Alpha-helix	Extended strand	Beta turn	Random coil
PsHSP20-1	24.53	19.50	5.66	50.31
PsHSP20-2	25.84	14.83	3.35	55.98
PsHSP20-3	29.41	12.94	3.53	54.12
PsHSP20-4	17.91	26.12	5.22	50.75
PsHSP20-5	29.17	21.53	5.56	43.75
PsHSP20-6	28.57	20.41	5.44	45.58
PsHSP20-7	20.43	13.04	3.48	63.04
PsHSP20-8	27.85	21.00	4.11	47.03
PsHSP20-9	26.58	20.25	4.43	48.73
PsHSP20-10	13.10	24.83	4.83	57.24
PsHSP20-11	24.07	19.75	4.32	51.85
PsHSP20-12	21.12	20.50	4.35	54.04
PsHSP20-13	28.57	19.25	4.35	47.83
PsHSP20-14	30.19	18.87	4.40	46.54
PsHSP20-15	27.39	19.11	5.10	48.41
PsHSP20-16	26.28	19.23	5.77	48.72
PsHSP20-17	22.74	11.91	2.53	62.82
PsHSP20-18	20.90	19.21	5.08	54.80
PsHSP20-19	17.67	20.14	3.18	59.01
PsHSP20-20	29.69	16.67	3.65	50.00
PsHSP20-21	27.08	17.19	3.65	52.08
PsHSP20-22	26.84	17.89	3.68	51.58
PsHSP20-23	29.05	20.95	5.41	44.59
PsHSP20-24	23.12	20.00	5.00	51.88
PsHSP20-25	27.41	21.48	6.67	44.44
PsHSP20-26	25.16	16.35	4.40	54.09
PsHSP20-27	18.94	24.79	5.01	51.25

PsHSP20-28	31.70	12.95	3.12	52.23
PsHSP20-29	19.28	16.87	4.22	59.64
PsHSP20-30	28.74	16.17	4.79	50.30
PsHSP20-31	13.54	18.34	3.93	64.19
PsHSP20-32	27.81	18.93	4.73	48.52
PsHSP20-33	25.47	19.88	4.35	50.31
PsHSP20-34	24.20	19.75	5.73	50.32
PsHSP20-35	19.15	21.99	5.67	53.19
PsHSP20-36	22.75	14.69	4.27	58.29
PsHSP20-37	26.00	18.00	4.67	51.33
PsHSP20-38	29.17	21.53	5.56	43.75
PsHSP20-39	26.01	13.00	3.14	57.85
PsHSP20-40	21.21	18.18	4.24	56.36
PsHSP20-41	21.30	17.16	3.55	57.99
PsHSP20-42	21.21	18.18	4.24	56.36
PsHSP20-43	21.21	18.18	4.24	56.36
PsHSP20-44	17.24	19.31	5.52	57.93
PsHSP20-45	19.41	12.66	4.22	63.71
PsHSP20-46	24.38	19.38	5.62	50.62
PsHSP20-47	21.25	20.00	5.62	53.12
PsHSP20-48	29.76	16.67	6.55	47.02
PsHSP20-49	21.25	20.00	5.62	53.12
PsHSP20-50	23.75	20.00	5.00	51.25
PsHSP20-51	24.32	16.22	5.41	54.05
PsHSP20-52	25.33	16.44	4.89	53.33
PsHSP20-53	22.96	24.44	7.41	45.19
PsHSP20-54	18.25	20.44	5.11	56.20
PsHSP20-55	24.50	17.50	4.00	54.00
PsHSP20-56	17.73	21.99	5.67	54.61
PsHSP20-57	23.30	18.18	4.55	53.98
PsHSP20-58	23.02	19.42	7.19	50.36

Supplementary Table S4 Chromosome localization information of PsHSP20s gene in peony

Gene	Chromosome number	Start position	Termination position	Template chain
PsHSP20-1	Chr01	206229431	206238816	-
PsHSP20-2	Chr01	295809924	295811231	-
PsHSP20-3	Chr01	318957183	318964003	+
PsHSP20-4	Chr01	809060843	809061279	+
PsHSP20-5	Chr01	1036969176	1036976656	+
PsHSP20-6	Chr01	1553883006	1553887032	+
PsHSP20-7	Chr01	1599226163	1599228288	-
PsHSP20-8	Chr01	1833695848	1833707339	-

PsHSP20-9	Chr01	1904259369	1904265520	+
PsHSP20-10	Chr01	2142097930	2142107512	+
PsHSP20-11	Chr01	2297691856	2297692344	-
PsHSP20-12	Chr01	2297701409	2297701894	-
PsHSP20-13	Chr01	2297921287	2297921772	-
PsHSP20-14	Chr02	20204425	20204904	-
PsHSP20-15	Chr02	22194124	22194597	-
PsHSP20-16	Chr02	22277407	22277877	+
PsHSP20-17	Chr02	485788013	485796851	+
PsHSP20-18	Chr02	552856106	552857827	-
PsHSP20-19	Chr02	554315270	554321318	+
PsHSP20-20	Chr02	986938423	986939001	-
PsHSP20-21	Chr02	987205195	987205773	-
PsHSP20-22	Chr02	987410449	987411026	+
PsHSP20-23	Chr02	1032745982	1032746428	+
PsHSP20-24	Chr02	1142477202	1142477684	-
PsHSP20-25	Chr02	1368495537	1368496013	-
PsHSP20-26	Chr02	1456581377	1456582368	+
PsHSP20-27	Chr02	1548641973	1548645823	-
PsHSP20-28	Chr03	109258048	109265472	+
PsHSP20-29	Chr03	639614410	639614910	-
PsHSP20-30	Chr03	645662568	645667145	-
PsHSP20-31	Chr03	1239960098	1239964073	+
PsHSP20-32	Chr04	203784061	203786462	-
PsHSP20-33	Chr04	446351105	446351590	+
PsHSP20-34	Chr04	1287165186	1287165659	-
PsHSP20-35	Chr04	1323186020	1323187018	-
PsHSP20-36	Chr04	1776869675	1776877333	-
PsHSP20-37	Chr04	2381539026	2381539794	+
PsHSP20-38	Chr05	14187181	14188411	-
PsHSP20-39	Chr05	308790538	308791733	+
PsHSP20-40	Chr05	898839688	898840185	+
PsHSP20-41	Chr05	898980878	898981387	+
PsHSP20-42	Chr05	899033181	899033678	+
PsHSP20-43	Chr05	899457533	899458030	+
PsHSP20-44	Chr05	1179748771	1179749208	+
PsHSP20-45	Chr05	1442301722	1442303002	-
PsHSP20-46	Chr05	1477307563	1477308045	-
PsHSP20-47	Chr05	1477580065	1477580547	-
PsHSP20-48	Chr05	1477623870	1477630043	-
PsHSP20-49	Chr05	1477983492	1477983974	-
PsHSP20-50	Chr05	1480110518	1480111000	+
PsHSP20-51	Chr05	1575039922	1575040652	-
PsHSP20-52	Chr05	1676993233	1676995212	-

PsHSP20-53	Chr05	1744880132	1744880608	-
PsHSP20-54	Chr05	2069461165	2069462124	-
PsHSP20-55	Chr05	2336169531	2336174721	+
PsHSP20-56	unchr_scaffold_3	59074553	59075454	+
PsHSP20-57	unchr_scaffold_267	83104452	83105741	+
PsHSP20-58	unchr_scaffold_947	1671932	1672351	-
